# Heavy metal leaching from wood ash before and after hydration and carbonation

**DOI:** 10.1007/s11356-024-33221-0

**Published:** 2024-04-16

**Authors:** Lisbeth M. Ottosen, Nina M. Sigvardsen

**Affiliations:** 1 DTU Sustain, Department of Environmental and Resource Engineering, Technical University of Denmark, 2800 Lyngby, Denmark; 2 The Concrete Laboratory, Danish Technological Institute, 2630 Tåstrup, Denmark

**Keywords:** Wood fly ash, Heavy metals, Leaching chemometric modeling, Soluble salts

## Abstract

Wood ashes can be used, e.g., as soil fertilizer or in construction materials; however, it is important to ensure that such use will not cause spreading of heavy metals and subsequent harm to the environment. Wood fly ashes (WFAs) generally have higher concentrations of heavy metals than wood bottom ashes. This paper focuses on the leaching of heavy metals from WFA, specifically identifying WFA characteristics that influence the leaching and changes in leaching caused by hydration and carbonation in ambient air. Chemometric modeling based on characteristics for eight different WFAs suggested that the leaching of Cr and Zn was associated with the concentration of K and the leaching of SO_4_^2−^, indicating a connection to the soluble K_2_(SO_4_) commonly found in WFAs. During the aging, both pH and conductivity of the WFAs decreased showing the formation of new phases. The leaching of Cd, Cu, Ni, and Pb was low initially and decreased to non-measurable after the aging. So did the leaching of Zn except from one of the WFAs. Thus, the part of the heavy metals, which were leaching originally, was built into the newly formed phases. The Cr leaching also showed a general decrease during the aging, however, not to similarly low levels. This means that the leaching Cr fraction was either not influenced by the aging processes or the formed phases contained water-soluble Cr. The continued leaching of Cr needs more attention as it may be the toxic and carcinogenic Cr(IV). As the chemistry and mineralogy of WFAs, inclusive of the mobility of the heavy metals, are subject to changes, increased knowledge on the chemistry determining these changes is needed to choose environmentally sound recycling options.

## Introduction

Worldwide, in 2022, the supply of wood fuel reached 1.97 billion m^3^, and in addition to this 46.4 million tons of wood pellets (World Bioenergy Association 2023). The wood fuel is combusted at very different scales, from private stoves to large, combined heat and power plants. A side effect of the combustion of wood and wood products is the generation of wood ashes. The ash content of biomass is the inorganic non-combustible fraction corresponding to the residue after a sample is completely burnt, and based on literature, Kleinhans et al. (2018) reported that the ash content in woody biomass was in the range of 0.5–7 wt% on a dry basis. The ash content is the absolute minimum of ash generated since the ash also will contain an organic fraction if the combustion is not complete, and, e.g., Smołka-Danielowska and Jabłońska (2022) reported that the ash generated was 2.6–18.3 wt% in small domestic wood-fired furnaces dependent on the wood fuel. Thus, the mass is reduced during the combustion, but the large numbers concerning the supply of wood fuel and pellets reported by World Bioenergy Association (2023) underline that it is vast masses of wood ashes that are generated.

Finding good recycling options for wood ash has gained much attention in research, and different options for utilization have been and are explored. For example, wood ashes as fertilizer have a long history in research, and already in 1875, the phosphorous content and the fertilizer value were discussed (Scientific American 1875), and still 150 years later, research is carried out, e.g. on the effects of wood bottom ash on soil characteristics and tree growth in a forest (Pitman et al. 2024). Other applications for the wood ashes could be as an amendment in composting (Kurola et al. 2011) or as raw material in the production of construction materials, e.g., in concrete as filler or aggregate replacement (Cheah and Ramli 2011; Carević et al. 2020a) or partial cement replacement (Berra et al. 2015). The European Green Deal stimulates the recycling of finite resources and underlines a zero-pollution ambition. In support of this, wood ashes should be considered a resource, but at the same time, their use should not cause environmental pollution.

Wood ashes contain heavy metals, which originate from the wood biomass combusted. During growth, the trees take up heavy metals from the soil. The total contents of heavy metal(loid)s in soil are the sum of the concentrations of elements derived from minerals in the geological parent material on which the soil has developed (lithogenic source) and inputs from a wide range of possible anthropogenic (contamination) sources (Alloway 2013). Thus, geographically and from site to site, the concentrations of heavy metals for potential uptake by the trees from the soil vary considerably. Even in rural areas, the forests have had an input of heavy metals. For example, an investigation of atmospheric heavy metal deposition to forests in the rural area of southern Scandinavia concluded that the accumulated atmospheric inputs over 50 years played a dominant role in the buildup of heavy metals in the top soils, and on the more positive side that the average concentrations of Pb, Cd, Cu, Zn, Ni and As in the aerosols all decreased from 1979/1980 to 2002/2005 (Hovmand et al. 2008). The actual uptake of heavy metals by the tree depends on the species (e.g., Mingorance et al. 2007), and the concentration between the compartments of a tree. For example, Grandois et al. (2010) found indications for Pb mainly being stored in the stem, whereas Zn and Ni, and to a lesser extent Cd and Cu, were translocated to aerial parts of the trees and cycled in the ecosystem for a forest stand in the North-eastern France. Another important source of heavy metals in wood fuel can be the aerosols deposited on the tree. Through a large sampling campaign, Reimann et al. (2001) found that, e.g., for pine, the concentration of some elements including heavy metals was significantly influenced by dust, whereas for other plants, dust played a less significant role. Altogether, the content of heavy metals in the wood fuel must be expected to vary significantly depending on, e.g., the local conditions during growth, the species of the tree, and the parts of the tree included in the fuel.

There is no EU-wide legislation regulating the content of the different heavy metals when used as soil fertilizer. Instead, various national laws and decrees state different limiting values; decrees for Denmark, Finland, and Sweden were compiled by Pesonen et al. (2017), and additionally for Lithuania, Austria, and Croatia by Carević et al. (2020b). Limiting concentrations for heavy metals in wood ash for use as in cementitious materials are also lacking, as no standard yet covers such use. Several researchers have reported that the heavy metal concentration is generally higher in the WFA than in the WBA (Steenari and Lindqvist 1997; Pitman 2006; Carević et al. 2020a). In Carević et al. (2020b) concentrations compiled from literature, WFAs, e.g., showed distinct different levels for Cd of and 0.4–0.7 mg Cd/kg in WBA and 1–60 mg/Cd in WFA and for Cu, the levels were for WFA 15–300 mg Cu/kg in WBA and 27–920 mg Cu/kg in WFA, i.e., the concentration levels were overlapping, while the highest concentrations were measured in WFAs. Thus, it is essential to distinguish between the two fractions concerning environmentally safe utilization. WFA can contain high concentrations of Cd, Cu, Cr, Pb, and As; accordingly, WFA ash should, e.g., not be used as fertilizer (Pitman 2006).

To find environmentally sound ways to utilize WFAs, it is essential to increase the knowledge on the behavior of the heavy metals, and not at least the mobility. Systematic studies about the concentration, modes of occurrence, behavior, and fate of hazardous phases and trace elements in biomass fuels, and their ashes are only at an initial investigation stage (Vassilev et al. 2013). The leaching characteristics of ashes provide useful information for selecting the appropriate management strategy and the possible use of wood ashes (Saqib and Bäckström 2014). However, it is essential to consider that the leaching properties will change from the ash leaving the combustion plant to the ash stored under ambient environmental conditions. During aging, a complex set of reactions occurs related to hydration and carbonation. The ash mineralogy changes as moisture and carbon dioxide from the air react with the oxides in the WFA to form carbonates, bicarbonates, and hydroxides (Etiégni and Campbell 1991). Using Ca salts as an example, the reactions are hydration (CaO + H_2_O → Ca(OH)_2_) and carbonation Ca(OH)_2_ + CO_2_ → CaCO_3_ + H_2_O) (Etiégni and Campbell 1991). The formation of gypsum (CaSO_4_·2H_2_O) and ettringite (Ca_6_Al_2_(SO_4_)_3_(OH)_12_·26H_2_O) has also been identified during the hydration (Steenari and Lindqvist 1997). Ettringite formation occurs in the presence of sulfate, soluble aluminate, and Ca(OH)_2_ (Steenari and Lindqvist 1997). The prevalence of ettringite or gypsum is determined by the content of Al in the specific wood ash (Sigvardsen et al. 2021). The changes in ash mineralogy during aging will influence heavy metal mobility. Currently, there is a lack of research on the effects of natural aging on the leaching properties of bioresource-derived fly ashes (Soininen et al. 2018), which is a focus concerning WFAs in the present investigation.

In support of the zero-pollution ambition in the European Green Deal, the state of the heavy metals in WFAs must be considered regardless of which recycling option is chosen. Both total concentration and mobility must be considered. Little is known about how the ash composition influences the leaching of heavy metals and the influence of aging on the mobility of heavy metals in WFAs. This paper experimentally investigates the leaching and desorption of heavy metals from different WFAs and the change in leaching after aging. The aim is to increase knowledge of the link between WFA characteristics and the leaching behavior of heavy metals under different conditions relevant to recycling.

## Materials and methods

### Investigated ashes

The investigation included eight WFAs from four Danish wood combustion facilities (WFA 1–4) and one Swedish (WFA5) (Table 1). The facilities from where the ashes were collected were chosen so the ashes represent the most common WFAs. Typical solid fuel combustion systems comprise three main types: grate, fluidized bed, and pulverized fuel combustion systems (Kleinhans et al. 2018). According to Lamers et al. (2018), grate combustion is predominately used for the combustion of biomass, whereas circulating fluidized bed combustion is gaining ground in, e.g., Austria, Canada, Denmark, Germany, Italy, and the Netherlands. Thus, ashes were selected from grate combustion and fluidized bed combustion. The grate-fired plants were chosen so they covered different fuels: wood chips (solely or cofired with wood powder) or wood pellets and different ranges of combustion temperatures (see Table 1).

**Table 1:** Overview of investigated WFAs with information on fuel (in bracket origin of the fuel), temperature, and combustion methods

	Fuel	Combustion method	Temperature (°C)
WFA1	Wood chips and powder (whole trees)	Grate	900–1000
WFA2	Wood chips and powder (whole trees)	Grate	1,000–1100
WFA3.1, WFA3.2	Wood pellets (logs)	Grate	1,000–1100
WFA4.1, WFA4.2	Wood chips (whole trees)	Grate	600–1000
WFA5.1, WFA5.2	Wood chips (whole trees)	Circulating fluidized bed	760–930

Two batches were sampled from three of the plants (WFA3-WFA5) at different sampling periods. The individual biomass combustion facilities supplied the WFA samples from their local storage. After sampling, the WFAs were stored in closed plastic buckets at 5 °C. All eight ashes were characterized, and the results were used in a multivariate analysis. One WFA from each plant was used for the other parts of the investigation.

### Characterization procedures

Characterization and extraction experiments were done with dried WFA (105  C, 24 h). The water content was measured as weight loss after 24 h at 105 ºC (calculated as weight loss over the weight of the wet sample). Al, Ca, K, P, Cd, Cr, Cu, Ni, Pb, and Zn concentrations in the WFAs were measured according to the US EPA 3015A method (U.S. EPA 2007), and the ICP-OES used was a Varian 720-ES. Leaching was done by suspending the WFA in distilled water at a liquid-to-solid (L:S) ratio of 2. After 24 h of agitation, the suspension was filtered, and the Cl^−^ and SO_4_^2−^ concentrations were measured in the filtrate by ion chromatography (Thermo Scientific Dionex ICS-1100), and the Al, Ca, Cd, Cr, Cu, Ni, Pb, and Zn concentrations with ICP-OES (Varian 720-ES). The L/S ratio of 2 for the leaching was chosen as the lowest L/S possible in practice to obtain a sufficient liquid phase from filtration, and a low L/S is preferable not to dilute the low leached concentrations of the heavy metals. The pH and conductivity were measured by suspending WFA in distilled water at an L/S ratio of 2.5 for 1 h and measuring the pH and conductivity with radiometer electrodes. Loss on ignition (LoI) after 30 min at 550 ºC was measured. Three to five replicates of each of these analyses were made. The WFA solubility in water was evaluated in two replicates after the water-washing procedure (Ottosen et al. 2013) and calculated as the percentwise loss in mass.

### Multivariate modeling

The present work uses chemometric modeling, and more specifically, a principle component analysis (PCA), to evaluate the influence of WFA characteristics as variables (Al, Ca, K, P concentrations, water content, pH, pH_HNO3_, solubility, leaching of Ca, Al, Cl^−^, and SO_4_^2−^, LoI, and the concentration of heavy metals) on the leaching of heavy metals as the responses. PCA reduces datasets with many interrelated variables while retaining the variation presented in the original data as much as possible. This reduction is achieved by transforming the actual variables into a new set of variables—the principal components. They are uncorrelated linear combinations of the variables. In Projections to Latent Structures (PLS), the quantitative relation between a descriptor matrix, *X*, and a response matrix, *Y*, is used to assess the comparative influence of descriptors on the responses and to predict responses at given descriptor settings within the studied experimental space (Wold et al. 2004). The SIMCA 16 (Sartorius Stedium Biotech) software was used for calculating PLS models. The models were evaluated from the correlation factor *R*^2^*Y* and the predictive power *Q*^2^. The *R*^2^*Y* is the cumulative fraction of the variation of the *Y* variable explained by the model after the last component and is a measure of fit, i.e., how well the model fits the data. The *Q*^2^ is the variation of the *Y* variable predicted by the model, after the last component, according to cross-validation. It is thus a measure of how well the model predicts data. A value of *R*^2^*Y* > 0.9 is excellent, while a value above 0.5 is good. The difference between *R*^2^*Y* and *Q*^2^ should be as low as possible, and a difference larger than 0.2–0.3 may indicate outliers or the presence of irrelevant variables in the *X* block (Carlson and Carlson 2005).

### The pH-dependent desorption

The desorption of heavy metals as a function of pH was investigated for one WFA from each plant. Closed plastic vials with WFA (1.0 g) and distilled water or HNO_3_ of different concentrations (25 ml) were agitated for 1 week. The HNO_3_ concentrations were 0.01 M, 0.05 M, 0.08 M, 0.1 M, 0.3 M, 0.5 M, 0.7 M, and 1.0 M. The pH was measured in the suspension with a pH electrode, and the suspension was filtered before the measurement of heavy metal concentrations in the filtrate by ICP-OES (Varian 720-ES).

### Size fractionation and heavy metals

The five WFAs were wet sieved into three fractions: < 0.063 mm, 0.063 <  ×  < 0.125 mm, and > 0.125 mm. Here, 100 g ash was mixed with distilled water to a slurry and first sieved through the 0.125-mm sieve. A rubber tool continuously moved the slurry on the sieve until no more passed. Then, the same procedure was carried out with the < 0.125 fraction on the 0.063 mm sieve. The < 0.063-mm fraction was first dried on a hot plate to evaporate the excess water, followed by drying in an oven at 105 °C. The two other fractions were dried in the oven at 105 °C. The heavy metal concentrations in each fraction were measured in the three fractions after the procedure described in paragraph 2.2.

### Hydration and carbonation

One WFA from each plant was carbonated in ambient air in the laboratory for the same period of 30 days. The procedure was to place 50-g WFA in a petri dish (diameter 100 mm), where after 100-ml distilled water was mixed with the ash by hand with a spatula. During the 30 days, additional distilled water was mixed into the ash about once a week to avoid drying out. Altogether, 300 g of extra distilled water was added. The pH, conductivity, and leaching were measured on the hydrated and carbonated WFAs.

## Results

### Characteristics of investigated ashes

The overall results from the characterization of the WFAs are in Table 2.

**Table 2:** Overview of characteristics of the investigated WFAs (– below the detection limit, *n.m.* is not measured)

	WA1	WA2	WA3.1	WA3.2	WA4.1	WA4.2	WA5.1	WA5.2
Cd (mg/kg)	6.7	11.5	14.6	6.0	9.2	11.1	12.9	9.6
Cr (mg/kg)	13	30	53	54	22	18	21	16
Cu (mg/kg)	90	150	140	93	160	99	67	72
Ni (mg/kg)	19	41	50	19	19	18	15	17
Pb (mg/kg)	30	38	23	41	66	43	28	16
Zn (mg/kg)	643	1960	1890	913	2050	1680	437	444
Ca (g/kg)	157	160	225	126	240	179	220	200
Al (g/kg)	6.8	7.7	4.7	6.9	7.4	8.3	9.9	13.8
P (g/kg)	7.2	17	14.1	15.9	16.5	8.9	13.6	15.3
K (g/kg)	63	84	86	88	103	57	27	32
Cd leaching (mg/kg)	0.02	-	-	0.17	0.01	0.33	-	0.11
Cr leaching (mg/kg)	4	15.2	32.8	20.5	12.5	3.3	1.8	2.2
Cu leaching (mg/kg)	0.13	0.01	0.08	0.01	0.01	0.03	0.01	0.01
Ni leaching (mg/kg)	0.09	-	0.09	-	0.02	-	0.02	-
Pb leaching (mg/kg)	0.17	0.13	0.43	0.06	2	0.22	0.24	0.05
Zn leaching (mg/kg)	1.93	8.7	24.9	0.08	40	9.2	-	0.05
Ca leaching (mg/kg)	303	152	242	130	315	514	1850	1270
Al leaching (mg/kg)	0.74	1.5	0.4	3.5	0.68	0.09	0.03	0.04
Cl^−^ leaching (g/kg)	21.4	18.9	5.1	4.9	3.0	6.8	4.5	2.6
SO_4_^2−^ leaching (g/kg)	50.4	46.3	57.3	52.4	66.4	30.6	16.3	5.1
Water content (%)	23.3	1.2	0.2	0.2	0.2	n.m	0.1	0.2
pH	12.8	13.3	13.1	12.4	13.5	13.3	12.7	12.8
pH_0.5 M HNO3_	5.2	11	12.5	1.1	7	3.1	4	2.9
Solubility (%)	14.8	24.1	15.3	13.3	13.4	11.6	6.2	4.9
LoI (550 °C) (%)	9.3	7.3	0.5	6.6	0.1	5.7	0.8	-

The WFAs were all alkaline, but all other characteristics varied considerably between the WFAs (Table 2). The LoI at 550 °C roughly expresses the unburned fraction of organic matter, and the range was from a not measurable content (WFA5.2) to 9.3% (WFA1). The water content (Table 2) was below 1.2% in all WFAs except WFA1, which was as high as 23.3% (due to spraying after the combustion to avoid dust for easier handling). The buffering capacity against acidification was evaluated through the pH measured 0.5 M HNO_3_ (pH_0.5MHNO3_) from the pH extraction experiment. The pH_0.5MHNO3_ varied significantly, from 1.1 for WA4.1 to 12.4 for WA4.2, showing a much higher buffering capacity of the latter. This difference underlines a major difference between WFA4.1 and WFA4.2 even though they were sampled at the same plant.

### Leaching of Cr and Zn and WFA characteristics

PLS was used to model the relation between the leaching of Cr and Zn and the WFA characteristics. The leaching of Cd, Cu, Ni, and Pb was too low and varied too little (Table 2) for a PLS model. The PLS model for Zn and Cr had the leaching as the response and the WFA characteristics (inclusive total concentrations of the same element) as variables. The quality of the models was evaluated from the correlation factor *R*^2^*Y* and the predictive power *Q*^2^. The (*R*^2^*Y*; *Q*^2^) for the two PLS models were Zn (0.986; 0.806) and Cr (0.798; 0.546), i.e., the model for Zn leaching was excellent, and the model for Cr leaching was good.

**Fig. 1:**
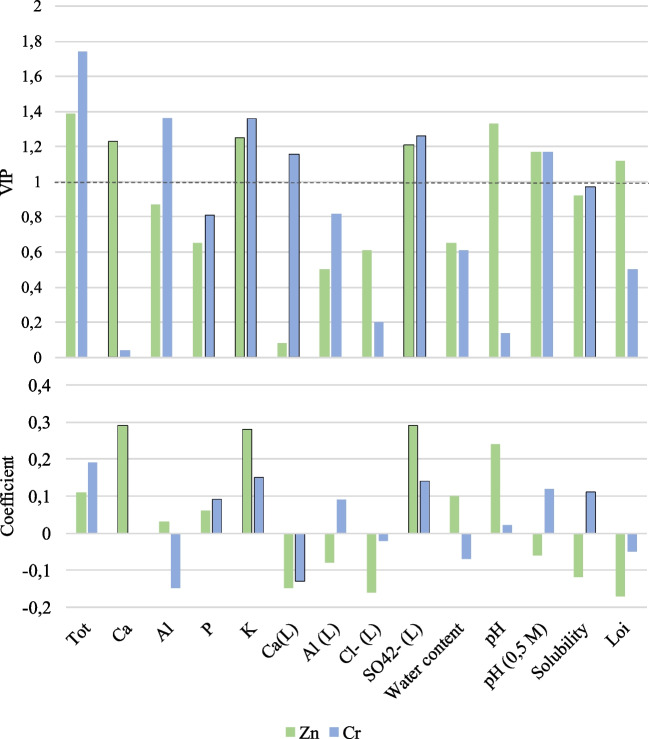
VIP and coefficient plots for the PLS models for leaching of Cr and Zn. Parameters with VIP values > 1 have high, 0.5–1 have moderate, and < 0.5 have low influence on the leaching. Parameters with coefficient values > 0 have a positive correlation, and coefficient values < 0 are oppositely correlated with the leaching

Variable importance in the projection (VIP) plots were made. VIP values present the absolute importance of each parameter in the model concerning its correlation to all the responses (*Y*) and the projection (*X*). VIP values > 1 represent a high influence of the variable(s), and VIP values < 0.5 indicate a low influence. VIP plots were used to assess the variable importance in the calculated models. Coefficient plots were used to evaluate if the variables had positive or negative impacts on the model responses. Confidence intervals (95%) on the coefficient indicate that the coefficient is significant (above noise) if the confidence interval does not include zero. Figure 1 summarizes the VIP plots and coefficients plots where the line for VIP = 1 is shown. The variables for which the coefficient showed significance to the 95% confidence interval are indicated by a solid line around the bar in the plots.

Variables with VIP > 1 and significance from the coefficient plots are (Fig. 1):

- Zn leaching was positively correlated to K concentration > Ca concentration > leaching of SO_4_^2−^
- Cr leaching was positively correlated to K concentration > leaching of SO_4_^2−^  > solubility and negatively correlated to leaching of Ca

### pH-dependent extraction

**Fig. 2:**
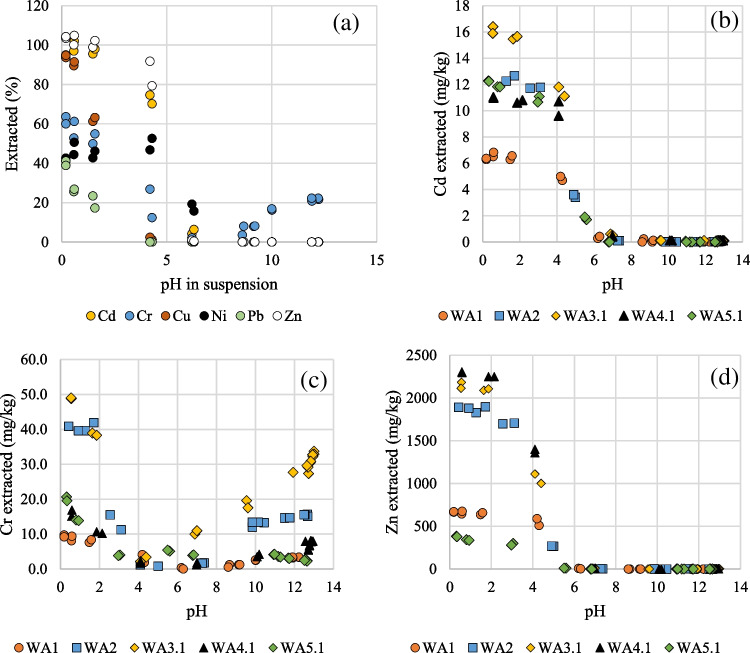
pH-dependent desorption of **a** all investigated heavy metals from WFA1 (in percentage), **b** Cd from five WFAs (in mg/kg), **c** Cr from five WFAs (in mg/kg), and **d** Zn from five WFAs (in mg/kg)

The desorbed percentages of the investigated heavy metals at different pH are shown for WFA1 in Fig. 2a. The extracted Cd, Cr, and Zn (mg/kg) from all WFAs at different pH are shown in Fig. 2b–d. From neutral pH towards increased acidification, the extraction increased with decreasing pH for all heavy metals. Cr was the only heavy metal extracted at neutral to alkaline pH, i.e., Cr was extracted at both high and low pH (highest at low pH).

The general trend for the desorption of heavy metals in all WFAs followed the same order when decreasing pH as WFA1 (Fig. 2a). The pH levels where desorption started: Ni (7–8) > Cd (6–7) > Zn (4–5) > Cu (~ 4) > Pb (~ 2). Cd, Cu, and Zn desorption approached 100% at a pH less than 1. The desorption of Pb and Ni at the lowest pH (< 0.5) was less and varied significantly between the ashes (31–95% Ni and 33–88% Pb).

### Cd, Cr, and Zn in different size fractions

**Fig. 3:**
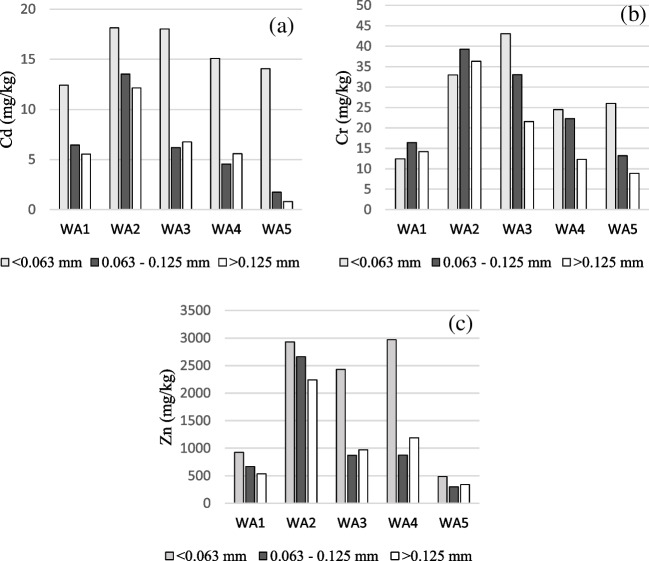
Concentrations in three size fractions **a** Cd, **b** Cr, and **c** Zn

The heavy metals are enriched in the WFA compared to the WBA, according to literature, e.g., (Lanzerstorfer 2017). It is interesting to investigate whether such enrichment of the heavy metals continues from the coarser to the finer fraction of the WFAs. The concentrations of Cd, Cr, and Zn in three fractions (< 0.063 mm; 0.063–0.125 mm; and > 0.125 mm) are seen in Fig. 3. The procedure for the fractionation was wet sieving, so the < 0.063-mm includes the water-soluble fraction. The Cd and Zn concentrations were highest in the < 0.063-mm fraction, and the concentrations in the two larger fractions were similar. The general pattern for Cr (Fig. 3b) was a more even distribution in the three fractions, which is probably linked to Cr being low volatile. The Cr concentration in the largest fraction (> 0.125 mm) was though less than in the 0.063–0.125 mm fraction in every WFA.

### Hydrated and carbonated WFAs

**Fig. 4:**
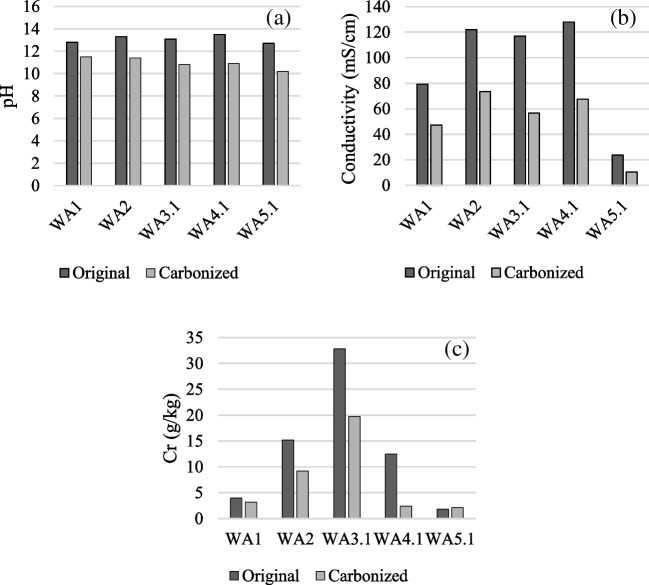
The **a** pH, **b** conductivity, and **c** leaching of Cr from the WFAs before and after hydration/carbonation in the lab

The pH and conductivity of the investigated WFAs decreased during the hydration/carbonation (Fig. 4a and b). WFA1, which initially had a high water content (23.3%), reacted similarly to the other ashes during the hydration/carbonation in the lab, revealing that full carbonation was not reached before WFA1 was delivered for this investigation. The carbonation temporarily stopped for WFA1 while stored in closed buckets.

After carbonation/hydration, Cd, Cu, Ni, Pb, and Zn leaching decreased to 0.02 mg/kg or less. The leaching of Cr from the WFAs before and after hydration/carbonation also decreased (a slight increase was found for WFA5.1); however, Cr leaching remained highest (2.1–19.7 mg Cr/kg) (Fig. 4c).

## Discussion

### Ash characteristics

The WFA characteristics varied considerably (Table 2), consistent with previously reported (Carević et al. 2019; Sigvardsen et al. 2019b). The characteristics depend on many factors: method of combustion, including temperature, species of tree, and practice of wood ash collection (Etiégni and Campbell 1991). None of the five combustion plants used the same fuel type, combustion method, and temperature range (Table 1); thus, variations in WFA characteristics are expected. Four of the WFAs (WFA1-4) originated from grate-fired plants, the most used type for biomass combustion (Lamers et al. 2018). The WFA5 originated from circulating fluidized bed combustion, which is increasingly used in many countries due to its high efficiency when only biomass is used as a fuel (Van Den Broek et al. 1996). Four of the five WFAs were from the combustion of fuels from whole trees, whereas the fifth (WFA3) was from the combustion of wood fuel from logs. The combustion temperature is essential to the WFA mineralogy. For example, a combustion temperature of 1000 ± 1200 °C, as in grate-fired boilers, often results in the formation of calcium silicates (Steenari et al. 1999). In contrast, the low temperature in the fluid bed combustion (850 °C) does not favor calcium silicates forming (Steenari et al. 1999). The differences in characteristics of the WFAs from the same combustion facilities (Table 2) must be ascribed to variations in fuel and incineration parameters on the specific days of sampling.

The main crystalline compounds formed during the combustion of wood are oxides, carbonates, sulfates, and chlorides (Steenari and Lindqvist 1997). The investigated WFAs contained sulfate and chloride salts (seen from the leaching of Cl^−^ and SO_4_^2−^). The content of both varied significantly: 5.1–66.4 g SO_4_^2−^/kg and 3.0–18.7 g Cl/kg. The solubility varied between 4.9% in WFA5.2 and 24.1% in WFA2 (Table 2). Previously, WFA from fluidized bed combustion was reported to consist of two distinctly different modes (Lind et al. 2000): a fine fly ash formed by nucleation of volatilized species (mainly KCl and K_2_SO_4_) and a coarse fly ash with irregular agglomerated particles formed from the non-volatile ash species by coalescence and agglomeration inside the char particles and on their surfaces. The coarser ash fraction (> 1 µm) was dominated by components containing non-volatile elements such as Ca, Si, Mg, Al, Fe, P, and Mn that have remained in the solid and liquid phases during the combustion process. The agglomerate structure of the coarse ash was effective in capturing volatile species in coarse particles (Lind et al. 2000).

### Heavy metals in the WFAs

The heavy metal concentrations in the investigated WFAs are all in the ranges reported in a literature study (Carević et al. 2020b) except for Ni, where the concentration range reported was 20–102 mg Ni/kg. The Ni concentrations in most of the investigated WFAs were slightly lower (Table 2). Limiting values for heavy metals in wood ashes for agricultural and forestry applications from different European countries (Finland, Sweden, Denmark, Lithuania, Austria, and Croatia) were compiled by Carević et al. (2020b). In general, Austria and Croatia have the lowest limiting values. The Cd concentration in all the investigated WFAs was higher than the Croatian limiting value (3 mg Cd/kg). Most WFAs also exceeded the Austrian limiting value (6 mg Cd/kg). The limiting concentrations for Zn in these two countries are 1800 and 1500 mg Zn/kg, respectively, and about half of the eight investigated WFAs complied with this. The concentrations of Cr, Cu, Ni, and Pb in the investigated WFAs meet the limiting values from all these countries.

The heavy metal distribution between WFA and WBA generally depends on the concentration in the fuel (Dahl et al. 2009), the combustion temperature and technology (Vassilev et al. 2010), and the content of Cl (Saqib and Bäckström 2014). WFA5 had the lowest Zn concentration (Table 2), consistent with Dahl et al. (2009), who found that Zn was enriched in the bottom ash and depleted from the fly ash in CFB compared to grate combustion. Model calculations for the chemical equilibrium of various heavy metals under biomass combustion suggested that Cd, Cu, and Pb should be efficiently volatilized during combustion and, to a lesser extent, Cr and Zn, whereas Ni should not be volatilized (Ljung and Nordin 1997). Thus, the relatively low temperature (Table 1) may be an important factor in the low concentration of Zn in the WFA5.1 and WFA5.2.

#### Leaching of Zn

Zn is the heavy metal with the highest concentration in the WFAs (440–2050 mg Zn/kg), and the Zn leaching ranges from non-measurable to 24.9 mg Zn/kg (Table 2). Under acidic conditions, Zn desorption starts at a pH of about 4–5 and reaches about 100% at a pH below 1 (Fig. 3d). The Zn concentration is highest in the finest fraction including the water-soluble (Fig. 4c), which is consistent with Lanzerstorfer (2015).

The leaching of Zn depends strongly on the K concentration and leaching of SO_4_^2−^ in the developed PLS model (Fig. 2). Arcintie (K_2_(SO_4_)) is the most commonly identified soluble salt K-containing in WFAs, e.g., Sigvardsen et al. (2019a), and the result from the PLS modeling indicates a link between a high arcintie concentration and the highest Zn leaching. Geochemical calculations by Mann and Deutscher (1980) reported that Zn(OH)_2_ and Zn_4_(SO_4_)(OH)_6_ were the solid phases that limit Zn solubility in most solutions containing carbonate, sulfate, and chloride ions. The dispersion solution from the WFA leaching contains these ions, but naturally, many others, which will also influence the phases; however, the geochemical model supports the finding from the PLS model of the leaching of Zn and SO_4_^2−^ being linked. Supporting this finding is also that in WFA3.1 and WFA4.1, the Zn concentration in the finest fraction with the water-soluble fraction was even more than twice as high as in the two other fractions (Fig. 4c), and these are the two WFAs with the highest concentrations of soluble SO_4_^2−^ (Table 2). A literature review (Vassilev et al. 2013) reported that alkali, chlorides, and sulfates and some trace elements (e.g., Zn) enriched in the finest fly ash fraction (< 1 µm) originated from volatilized elements that nucleate to form new particles or condense on existing particles during the flue gas cooling. Since the fine fraction of the current investigation covers (< 63 µm), the concentration difference between the fractions would probably have been even larger if only smaller grain sizes had been in the fine fraction.

The Zn leaching was positively correlated to the Ca concentration, the pH, and the pH_0.5 M_ (Fig. 2). This could be due to the co-precipitation of Zn in carbonates, but no conclusion can be drawn based on this.

#### Leaching of Cd

Cd is generally the heavy metal that causes the most concern for its potential bioavailability and toxicity. The Cd leaching from the investigated WFAs ranged from not measurable (three WFAs) to 0.33 mg Cd/kg (WFA4.2) (Table 2). On this basis, a PLS model for Cd could not be made. The Cd desorption started at a relatively high pH of about 6–7, i.e., before the other investigated heavy metals except Ni. The Cd concentration was highest in the fine fraction of the WFAs (Fig. 4a), corresponding to Cd being highly volatilized during the combustion. The highest Cd concentration was also reported in the fine fraction by Dahl et al. (2010). In Lanzerstorfer (2017), the largest enrichment factors for heavy metals from WBA to WFA were those for Cd and Zn.

#### Leaching of Cr

The Cr leaching from the WFAs was relatively high compared to the other heavy metals (Table 2). Cr is in the middle group of volatile heavy metals together with Zn (Ljung and Nordin 1997). The Cr concentration is contrary to Zn and Cd, not consistently highest in the finest fraction of the WFAs (Fig. 4b). Cr also differs in being desorbed at both high and low pH (Fig. 3c), which is in accordance with the literature (Pöykiö et al. 2009; Maresca et al. 2017). The oxidation state for Cd, Cu, Ni, Pb, and Zn in the environment is + 2, whereas trivalent and hexavalent Cr may be found. Due to the prevailing oxidizing conditions, most constituents are present in their oxidized forms, e.g., Cr-III is partly converted into Cr-VI (Pohlandt-Schwandt et al. 2002). The Cr speciation changes over the pH range. In an aquatic environment in the absence of complexing agents other than H_2_O or OH^−^, the following trivalent Cr(III) species may be found (Ščančar and Milačič 2014): pH < 4.0 Cr^3+^, in less acidic solution, there are Cr(OH)^2+^ and Cr(OH)_2_^+^ species, and in the neutral up to alkaline pH region, Cr^3+^ is mainly precipitated as a sparingly soluble Cr(OH)_3_(s); however, in alkaline solutions at pH higher than 11.5, the precipitate re-dissolves, resulting in the formation of Cr(OH)_4_^−^ complex. The hexavalent Cr(VI) species present in aqueous solutions are H_2_CrO_4_ (pH < 1), HCr_2_O_7_^−^ pH (1–6.5), and CrO_4_^2−^ (pH > 6.5) (Ščančar and Milačič 2014). Due to this complexity, it is not possible to conclude the oxidation state of Cr from the pH desorption experiment. Nevertheless, it is necessary to consider the oxidation state since the Cr(VI) compounds are toxic and carcinogenic. In, e.g., Germany, there is a limit value for Cr(VI) in wood ash for application on arable land (Bachmaier et al. 2021). The level of Cr(VI) in wood ash is debated, and the authors of Bachmaier et al. (2021) recommend paying particular attention to Cr(VI) during the recycling of wood ash.

The PLS model revealed that the Cr leaching was positively correlated to K concentration and the leaching of SO_4_^2−^, and thus as for Zn, it indicates that a high arcintie concentration in the WFAs correlates to a higher leaching of Cr. The Cr leaching also correlates to the solubility (Fig. 2). This might be due to part of the Cr being precipitated with the soluble salts or the soluble salts influencing the particles with Cr when the WFA was suspended in water. It supports the relation to the arcentie content. The PLS model also revealed that the leaching of Cr was negatively correlated to the leaching of Ca. This indicates that the positive correlation between the Cr leaching and the solubility does not include WFAs with a high content of soluble Ca salts. There was an indication of the Cr leaching to be positively correlated to the P and Al concentrations and the pH_0.5 M_ (Fig. 2), though the correlation to Al concentration and buffering capacity was both influenced by noise compared to the 95% confidence interval, and the VIP value for the P concentration was less than 1. In summary, the leaching of Cr is highly complex, and it is strongly influenced by the chemical composition of the WFA, not at least the soluble salts.

### Changes caused by hydration/carbonation

#### Ash mineralogy

Hydration and the following carbonation of the ashes caused a pH decrease (Fig. 4a) from the reactions described in the introduction. The water content (Table 2) was below 1.2% in all WFAs except WFA1 at 23.3%. Ettringite formation and other hydration reactions in ash can also be initialized by water absorption from humid air (Steenari and Lindqvist 1997; Sigvardsen et al. 2019a). Thus, all WFAs may have changed from just leaving the boiler to sampling for the current investigation. The changes in pH and conductivity (Fig. 4a and b) show that reactive phases were still present in all WFAs.

During the hydration, ettringite formation is facilitated by a higher Al content, whereas a lower Al concentration promotes the formation of gypsum (Sigvardsen et al. 2021). The Al concentration varied from 4.7 to 9.9 mg/kg in the investigated ashes (Table 2), and a difference in ettringite and gypsum formation between the WFAs must be expected. The decrease in pH from carbonation likely caused additional changes in the ash mineralogy. For example, in an aqueous system, measurements performed in non-equilibrium conditions showed that the boundary for the disappearance of ettringite is pH = 10.7 and for monosulfate pH = 11.6 (Gabrisová et al. 1991), i.e., at pH values around those found in the hydrated and carbonated WFAs (Fig. 4a). At lower values of pH, only gypsum and aluminum sulfate were present in the system studied (Gabrisová et al. 1991). Figure 4b shows that conductivity also decreased by hydration/carbonation, revealing that new phases, which were not soluble in water, were formed.

#### Heavy metal leaching

The leaching of Cd, Cu, Ni, Pb, and Zn after hydration/carbonation decreased to non-measurable in all WFAs except for WFA2 for Zn (0.02 mg/kg). This reveals that they were built into newly formed phases, which were not soluble in water. For example, in ettringite, a substitution with Ca in with the divalent heavy metal ions has been reported (Gougar et al. 1996). The solubility curve predicted through a geochemical simulation suggested that smithsonite (ZnCO_3_) was important in controlling the leaching of Zn from aged cement-solidified MSWI fly ash (Du et al. 2018), and smithsonite might also be important in the decreased Zn leaching from the investigated WFAs after hydration/carbonation.

The leaching of Cr also decreased from all WFAs, but still leaching from 2.1 to 19.7 mg Cr/kg was seen in the hydrated/carbonated WFAs (Fig. 4c). Previously, Cr-bearing carbonates were identified in biomass ashes (Vassilev and Vassileva 2020). This investigation showed that the Cr leaching from the ash before hydration/carbonation was negatively correlated to the Ca-leaching, i.e., the higher the Ca-leaching, the less Cr leaching. Thus, the decreased Cr leaching might be linked to the hydration/carbonation from CaO to CaCO_3_; however, the continued leaching indicates that part of the Cr was present in compounds not influenced by the hydration/carbonation reactions. This points to the possibility of Cr(VI) still being present; however, this needs further investigation.

### Heavy metals and utilization of WFA

The lack of internationally agreed limiting concentrations and the different levels of the national limiting concentrations for heavy metals in WAs used as fertilizer means that, e.g., all the investigated WFAs could be spread in countries such as Sweden, Finland, and Denmark, while not in Croatia and Austria. This shows that not all the national limiting concentrations, if any, are based solely on an objective scientific evaluation of the environmental consequences of this utilization. Related to use in concrete, there are yet no limiting concentrations, as standards do not allow for such use; however, the intensive research and the European circular economy strategy of increasing the recycled content in products (European Commission 2020) may change this practice, which also calls for internationally agreed limiting concentrations, which are based on objective knowledge on, e.g., the mobility of the heavy metals. To protect the environment, deciding on limiting concentrations for heavy metals in WFAs related to the different uses should take into account the changes in ash chemistry and mineralogy and the subsequent influence on heavy metal mobility during the actual use. For example, the total metal content in soils is not generally a good predictor of metal solubility; the actual soil chemistry plays a major important role (McBride et al. 1997). Research and implementation work is urgently needed to elucidate dosage-dependent biological outcomes of bioash amendments, especially those related to soil and aquatic microbiomes as the primary living barriers/biofilters for most substances released from bioashes (Ondrasek et al. 2021).

The current paper adds to the necessary knowledge and underlines that the mobility of the heavy metals in the ash obtained from the combustion facility depends on the characteristics of the ash, and strong indications that the leaching of both Zn and Cr depends on the soluble salt were seen. When recycled, the chemistry changes due to the changed environmental conditions. Decreasing pH to about 4 resulted in a substantial increase in desorption of all the investigated heavy metals except Pb, and Cr also desorbed at alkaline pH, which is in accordance with the literature (Maresca et al. 2017; Ondrasek et al. 2021). It was also found that the leaching of all heavy metals decreased during hydration/carbonation, though Cr leaching was not to the same high extent as the other heavy metals. The mechanism of this decrease needs to be understood to evaluate the stability of the newly formed compounds or minerals under new or changing environmental conditions. The chemistry and mineralogy of WFAs inclusive mobility of the heavy metals changes with changing conditions; this must be addressed when choosing recycling options.

## Conclusions

The leaching of Cr and Zn from eight WFAs had a magnitude that enabled a principal component analysis focusing on connections between leaching and selected characteristics. The leaching of both Cr and Zn depended on the K concentration and leaching of SO_4_^2−^, indicating that a leachable fraction is connected to the soluble salts, where K_2_(SO_4_) is known to be common. After aging (hydration followed by carbonation in ambient air in the laboratory), the Zn-leaching was non-measurable in four WFAs and decreased from 8.7 to below 0.02 mg Zn/kg in the fifth. Thus, the leaching significantly decreased, i.e., Zn originally in the soluble fraction was built into the newly formed phases. Likewise, Cd, Cu, Pb, and Ni leaching decreased to a non-measurable level. The Cr leaching also showed a general decrease during the aging, however, not to similarly low levels. The Cr leaching was 2.1–19.7 mg Cr/kg compared to 1.8–32.8 mg Cr/kg before the hydration/carbonation. The leaching Cr fraction is either originally leachable Cr compounds that were not influenced by the hydration/carbonation or newly formed soluble phases with Cr. The investigation showed that the leaching of heavy metals decreases when WFAs undergo aging, which is encouraging related recycling. However, the stability in different environments of the newly formed phases with heavy metals must be investigated before conclusions can be drawn, and the continued leaching of Cr needs more attention as it may be the toxic and carcinogenic Cr(IV).

## Data Availability

The data that support the findings of this study are available from the corresponding author, LMO, upon reasonable request.
